# Supplementary material for: The histone code of the fungal genus Aspergillus uncovered by evolutionary and proteomic analyses
Source: Microb Genom. 2022 Sep 21;8(9):mgen000856. doi: 10.1099/mgen.0.000856 (PMC9676040; doi:10.1099/mgen.0.000856)
Supplement: Supplementary material 2 [file mgen-8-856-s002.pdf]

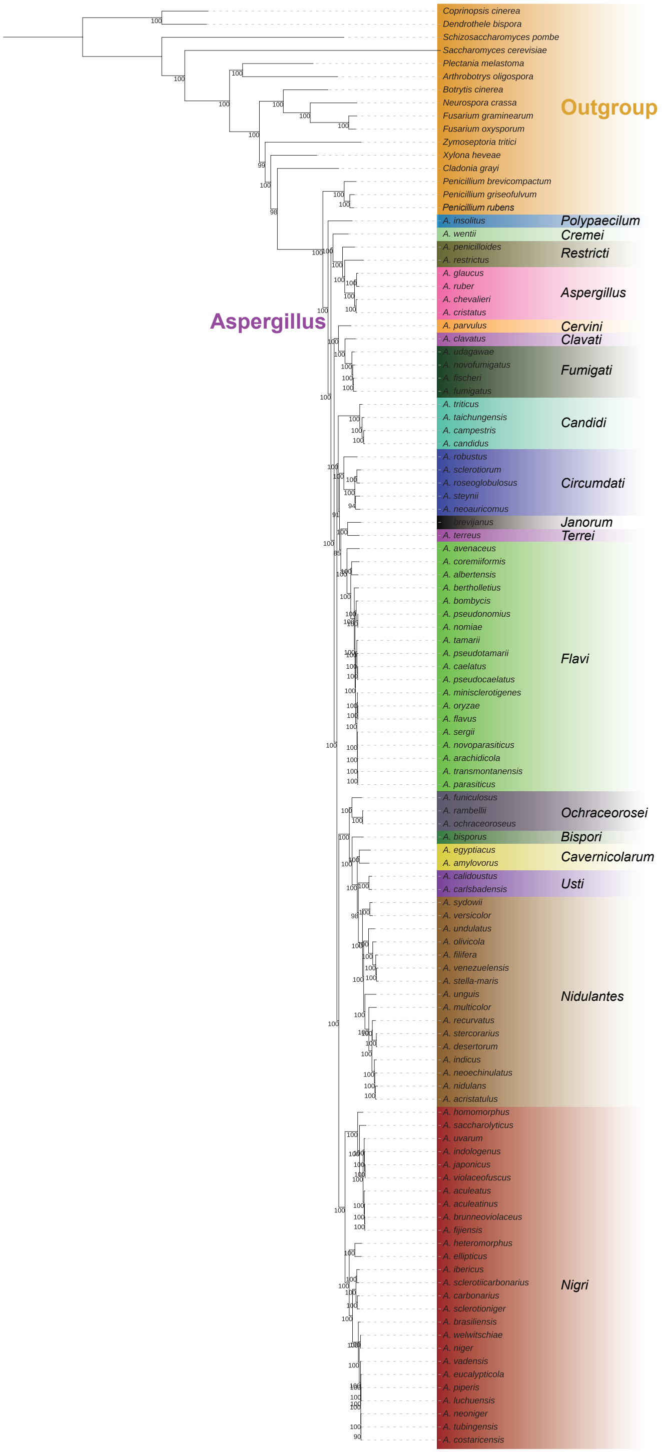

Supplementary Figure 1. A robust species phylogeny for the fungal genus *Aspergillus*. The species phylogeny was based on a maximum-likelihood phylogeny reconstructed by IQ-TREE with partitioned analysis using 758 BUSCO (Benchmarking Universal Single-Copy Orthologs) genes. The dot on the branch indicates ultrafast bootstrap values over 95 and SH-aLRT bootstraps values over 80. Species from the same section are labeled by background color with a section name. Two species, *Fusarium oxysporum* and *Aspergillus kawachii*, are included in the species tree, but we don't use these two in the following analyses.

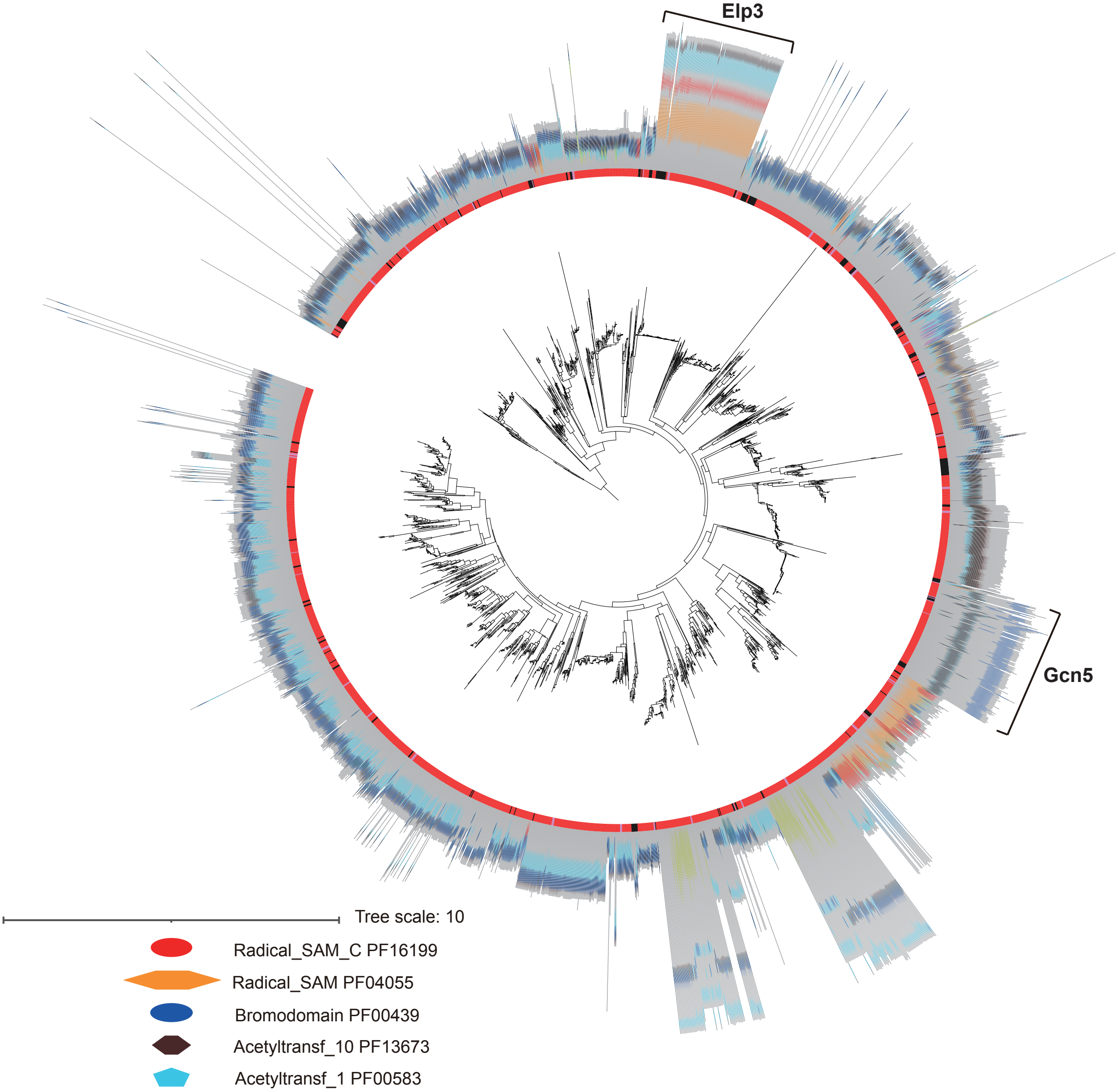

Supplementary Figure 2. Two known histone acyltransferase-domain containing protein groups are conserved in the *Aspergillus* genus and outgroup species together with many unknown proteins. Maximum likelihood phylogeny of Histone acyltransferase domains (PF00583) found in 94 Aspergilli and 15 outgroup species were determined using IQ-TREE (Chernomor et al. 2016; Nguyen et al. 2015). The black dot on the branch indicates ultrafast bootstrap values over 95 and SH-aLRT bootstraps values over 80. Additional conserved domains in histone acyltransferase domain-containing proteins were identified using the PFAM database (https://pfam.xfam.org).

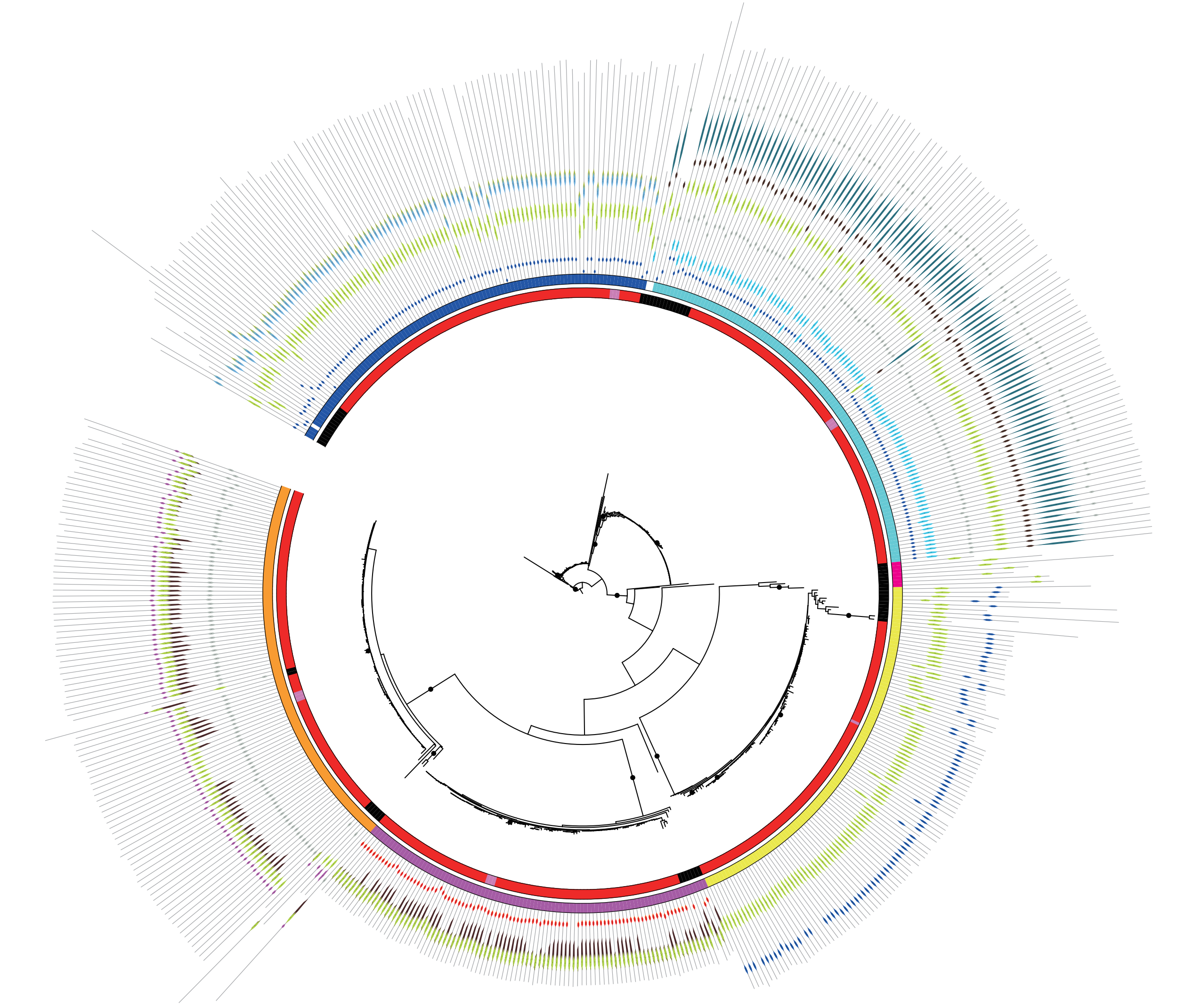

Tree scale: 1

- JmjC PF02373
- zf-HC5HC2H PF13771
- JHD PF17811
- Cupin_8 PF13621
- PLU-1 PF08429
- zf-C5HC2 PF02928
- zf-HC5HC2H_2 PF13832
- ARID PF01388
- PHD PF00628
- JmjN PF02375
- F-box-like PF12937
- zf-4CXXC_R1 PF10497

Group	Function
KDM4A/JHDM3/JMJD2	Demethylate H3K9/K36me2/3
Unknown	Unknown
KDM5/JARID	Demethylate H3K4me2/3
KDM6B/JMJD3	Demethylate H3K27me2/3 in human
DMM-1	Demethylase activity is not detected yet in *Neurospora crassa*
JMJD6/PKDM11	Demethylate H4R3me2 in Arabidopsis
KDM2/Epe1	Demethylate H3K36me1/2 in mouse, no demethylase activity detected in *N. crassa*

Supplementary Figure 3. Five histone demethylase-domain containing protein groups are conserved in *Aspergillus* genus and outgroup species while KDM6B/JMJD3 is lost. Maximum likelihood phylogeny of Jumonji C (JmjC) domain (PF02373) found in 94 Aspergilli and 15 outgroup species were determined using IQ-TREE (Chernomor et al. 2016; Nguyen et al. 2015). The black dot on the branch indicates ultrafast bootstrap values over 95 and SH-aLRT bootstraps values over 80. Additional conserved domains in JmjC domain-containing proteins were identified using the PFAM database (https://pfam.xfam.org).
